# ERK1/2 and HIF1*α* Are Involved in Antiangiogenic Effect of Polyphenols-Enriched Fraction from Chilean Propolis

**DOI:** 10.1155/2015/187575

**Published:** 2015-08-10

**Authors:** Alejandro Cuevas, Nicolás Saavedra, Martina Rudnicki, Dulcineia S. P. Abdalla, Luis A. Salazar

**Affiliations:** ^1^Center of Molecular Biology and Pharmacogenetics, Scientific and Technological Bioresource Nucleus, Universidad de La Frontera, Avenida Francisco Salazar 01145, 4781218 Temuco, Chile; ^2^Department of Clinical and Toxicological Analysis, Faculty of Pharmaceutical Sciences, University of São Paulo, Avenida Professor Lineu Prestes 580, 05508-000 São Paulo, SP, Brazil; ^3^Departamento de Ciencias Preclínicas, Facultad de Medicina, Universidad de La Frontera, Claro Solar 115, 4781218 Temuco, Chile

## Abstract

Propolis has been shown to modulate the angiogenesis in both *in vitro* and *in vivo* models. Thus, we aimed to evaluate the antiangiogenic properties of an ethanolic extract of Chilean propolis (EEP) and Pinocembrin (Pn). Migration, formation of capillary-like structures of endothelial cells, and sprouting from rat aortic rings were used to assess the antiangiogenic properties of EEP or Pn. In addition, microRNAs and VEGFA mRNA expression were studied by qPCR. ERK1/2 phosphorylation and HIF1*α* stabilization were assessed by western blot. EEP or Pn attenuated the migration, the capillary-like tube formation, and the sprouting in the *in vitro* assays. In addition, the activation of HIF1*α* and ERK1/2 and the VEGFA mRNA expression was significantly inhibited in a dose-dependent manner. In summary, these results suggest that HIF1*α* and ERK1/2 phosphorylation could be involved in the antiangiogenic effect of Chilean propolis, but more studies are needed to corroborate these findings.

## 1. Introduction

Propolis is a polyphenol-rich resinous substance produced by honeybees (*Apis mellifera*) from exudates of trees and plants which they use to seal holes in the beehive [1]. Its composition is very complex and varies according to climate, flora, and phenology of the geographical area where it was collected [2]. It has been shown that extract of propolis exhibits several biological activities such as antibacterial [3], antifungal [4], anti-inflammatory [5], antioxidant [6], anticancer [7], and antiatherogenic [8] properties.

Compelling evidence has shown that polyphenol-enriched fraction from propolis can modulate angiogenesis in both* in vitro* and* in vivo* models [8–13]. Angiogenesis is the physiological process through which new blood vessels emerge from preexisting vessels [14]. During postnatal and adult life, angiogenesis is the only mechanism that allows the formation of new blood vessels and is key in wound repair, female reproductive cycle, and exercising muscle [15]. By contrast, imbalance between activating and inhibitory factors of this process leads to pathological angiogenesis, persistent condition involved in tumor growth and progression [16], chronic inflammatory diseases such as Crohn's disease [17], cartilage destruction in rheumatoid arthritis [18], blindness in diabetes [19], growth of atherosclerotic plaques [20], and many other pathological processes.

The molecular mechanisms involved in the antiangiogenic effect of propolis are poorly understood. Furthermore, the demonstrated mechanisms are varied and likely depend on the particular composition of the extract used; so previously reported results cannot necessarily be extrapolated to other extracts. Moreover, not all studies have clarified whether extract concentrations used do not produce a cytotoxic effect. In this regard, the possible* in vitro *antiangiogenic effect of the Chilean propolis extracts has not been studied.

In the present work, the possible* in vitro *antiangiogenic activity of both ethanolic extracts of Chilean propolis (EEP) and Pinocembrin (Pn), one of its main constituents, was evaluated, at no toxic and no apoptotic concentrations.

## 2. Materials and Methods

### 2.1. Preparation of Ethanolic Extract of Chilean Propolis

Crude brown propolis was obtained from a mountainous area (latitude −38° 58′ 40.46′′, longitude −72° 1′ 15.73′′) near Cunco city,* La Araucanía*, Chile. The EEP was performed as previously described [8]. Briefly, crude propolis was mixed with ethanol 80% in a 1 : 3 w/v proportion in an amber colored bottle and then incubated for 30 min at 60°C under constant mixing. Then, the mixture was filtrated on a Whatman No. 1 filter paper in order to separate the ethanolic extract from crude propolis residues. For one night, the extract was left at 4°C, in order to promote the precipitation of waxes and other poorly soluble waste, and then centrifuged. Subsequently, the EEP was lyophilized and reconstituted in a 2 : 1 w/v proportion with DMSO. Finally, the EEP was quantified by Folin-Ciocalteu method and diluted at 50.000 *μ*g of gallic acid equivalent/mL (onwards expressed as *μ*g/mL) with DMSO for subsequent experiments.

### 2.2. Cell Culture

Human umbilical vein endothelial cells (HUVECs) were maintained in growth medium RPMI 1640 (GIBCO, Germany) at 37°C in a humidified atmosphere of 5% CO_2_ in air. The medium was supplemented with 10% heat inactivated fetal bovine serum (FBS), 100 IU/mL penicillin, and 100 *μ*g/mL streptomycin. Before each experiment the supplemented growth medium was replaced with medium supplemented with 1% FBS and incubated for 12 h.

### 2.3. MTT Viability Assay

The MTT reduction assay was done in 96-well plates at a density of 5 × 10^3^ HUVECs per well after treatment of HUVECs with different concentration of EEP or Pn. MTT 5 mg/mL in PBS was added to the culture medium at a final concentration of 0.5 mg/mL. After 4 h of incubation the reduced formazan was solubilized with DMSO and the absorbance measured at 570 nm in a microplate reader (Synergy MX, Biotek Instruments, USA).

### 2.4. Annexin V-FITC/PI Staining Experiment

Apoptotic cells were measured with an* Annexin V-FITC Apoptosis Detection Kit *(Sigma-Aldrich, USA) according to the manufacturer's protocol. Briefly, confluent HUVECs monolayers were treated with different concentration of EEP or Pn for 24 h at 37°C. Cells were then harvested and resuspended in the 1x-binding buffer. Cells were stained with 10 *μ*L Annexin V-FITC and 5 *μ*L propidium iodide (PI) for 15 min at room temperature in the dark. Analysis was performed by flow cytometry (FACS Canto, BD Biosciences, CA, USA) to identify the subpopulations of the apoptotic cells.

### 2.5. Cell Cycle Analysis

The ratio of cells in the G0/G1, S, and G2/M phases of cell cycle was determined by their DNA content. In 6-well plates cells at concentration of 2 × 10^5^ cells per well were treated with various concentrations of EEP or Pn for 24 h. Then, cells were harvested, transferred to cytometry tube, and centrifuged. Then, 200 *μ*L of lysis buffer (0.1% sodium citrate, 0.1% Triton), 20 *μ*L of RNAse A (Invitrogen, USA), and 2 *μ*L (1 mg/mL) of propidium iodide (Sigma-Aldrich, Steinheim, Germany) were added and were incubated for 30 min at 37°C and analyzed by flow cytometry.

### 2.6. Migration Assay

HUVECs migration was analyzed using an* in vitro* scratch wound assay as previously described [21]. In brief, confluent HUVECs monolayers were scratched with a sterile pipette tip, rinsed, and incubated for 8 hours with RPMI 1% FBS. The wounding area was photographed every 2 hours, up to a total of 8 hours. The TScratch software [22] was used to determine the extent of migration by quantifying uninvaded area in 3 distinct microscopic fields representative of each culture plate. Each experiment was performed in triplicate and repeated 3 times. The relative migration was expressed as

(1) $$\begin{array}{c} \left(\;\frac{{WA}_{t0}-{UA}_{tx}}{{WA}_{t0}}\;\right)\times 100, \end{array}$$

where WA_*t*0_ = wound area at time 0; UA_*tx*_ = uninvaded area at *x* time.

### 2.7. Tube Formation Assay

The capillary-like formation assay was performed as described previously [23], with slight modifications. Matrigel (BD Biosciences, CA, USA) was thawed at 4°C overnight. 50 *μ*L of Matrigel was added to each well of the 96-well culture plates and was allowed to polymerize at 37°C for 30 min. The HUVECs, to be tested for tube formation, were detached from the tissue culture plates, washed, resuspended in RPMI 1640 medium containing 1% FBS (8 × 10^3^ cells/well), and then added to the Matrigel-coated wells with various concentrations of EEP or Pn in the presence of VEGFA 10 *μ*g/mL. The plates were incubated at 37°C for 6 h in 5% CO_2_. After incubation, the capillary-like tube formation of each well in the culture plates was photographed with a Nikon light microscope. Each experiment was performed with 2 replicates each time and repeated 3 times. The angiogenesis score was calculated considering the number of sprouting cells, the number of connected cells, number of polygons, and complexity of the formed mesh according to the formula described by Aranda and Owen [24].

### 2.8. Aortic Ring Assay

Dorsal aorta from a 2-month-old male Wistar rat was taken out in a sterile manner and rinsed in ice-cold PBS. It was then cut into ~1 mm long pieces using surgical blade. Each ring was embedded in 3-dimensional rat collagen I gels (2 mg/mL) in 48-well plate and overlaid with 1.2 mL MCDB131 medium containing VEGF 10 *μ*g/mL, with or without 15 *μ*g/mL of EEP or Pn. On day 6, the rings were photographed and capillary-like structures were quantified. Each experiment was performed with at least 5 samples each time and repeated 3 times.

### 2.9. ERK1/2 Phosphorylation and HIF1*α* Stabilization

The western blot analysis was performed as previously described [25]. Briefly, cells for the study of HIF1*α* factor were treated with different concentrations of EEP and Pn and incubated for 4 h in a hypoxia chamber (air replaced by nitrogen gas), reaching concentrations below 1% oxygen. Meanwhile, the cells used for the study of ERK1/2 phosphorylation were incubated for 15 min in standard conditions from the application of VEFG 10 ng/mL stimulus. Treated cells were washed with ice-cold PBS, lysed with RIPA buffer (Sigma-Aldrich, Steinheim, Germany), scraped off, and sonicated followed by centrifugation (15,000 ×g, 15 min). Protein content was quantified and 100 *μ*g of total protein was loaded on 10% SDS-polyacrylamide gels and blotted onto nitrocellulose membranes. Nonspecific binding was blocked with 5% (w:v) defatted milk powder in TTBS for 1 h followed by antibodies incubation with HIF1*α* or ERK1/2 and pERK1/2 (1 : 1000 in 1% TTBS) overnight at 4°C. Blots were then incubated with goat anti-mouse antibodies conjugated to HRP (1 : 2000 in 1% TTBS) for 1 h followed by chemiluminescence detection. Band intensities were quantified by using ImageJ 1.48 software (NIH, USA).

### 2.10. mRNA and miR Expression

HUVECs cells (4 × 10^4^ cells/well) seeded in 12-well plates were incubated in media containing 10 *μ*g/mL of EEP or Pn for 24 hours. Cells were then lysed and the total RNA was isolated by using TRIreagent RNA isolation reagent (Ambion, USA) according to the manufacturer's instructions. Total RNA enriched with miRNAs was isolated by using or mirVana miRNA isolation kit (Life Technologies, USA). RNA was reverse-transcribed by High Capacity RNA to cDNA master mix (Life Technologies, USA). For microRNAs reverse transcription was used stem loop primer provided by the microRNA assay's manufacturer (Life Technologies, USA). All real-time PCR were performed using Power SyBR Green master mix (Life Technologies, USA) and analyzed with QPCR application [26].

### 2.11. Statistical Analysis

All the experiments were repeated at least three times. The results were expressed as mean ± S.D., and the data were analyzed using one-way ANOVA followed by Dunnett's test or Student's *t*-test using Sigma Plot (Sigma Plot for Windows, version 10.0, USA) to determine any significant differences. *P* < 0.05 was considered statistically significant.

## 3. Results

### 3.1. Cell Viability, Apoptosis Detection, and Cell Cycle Assays

**Figure 1:**
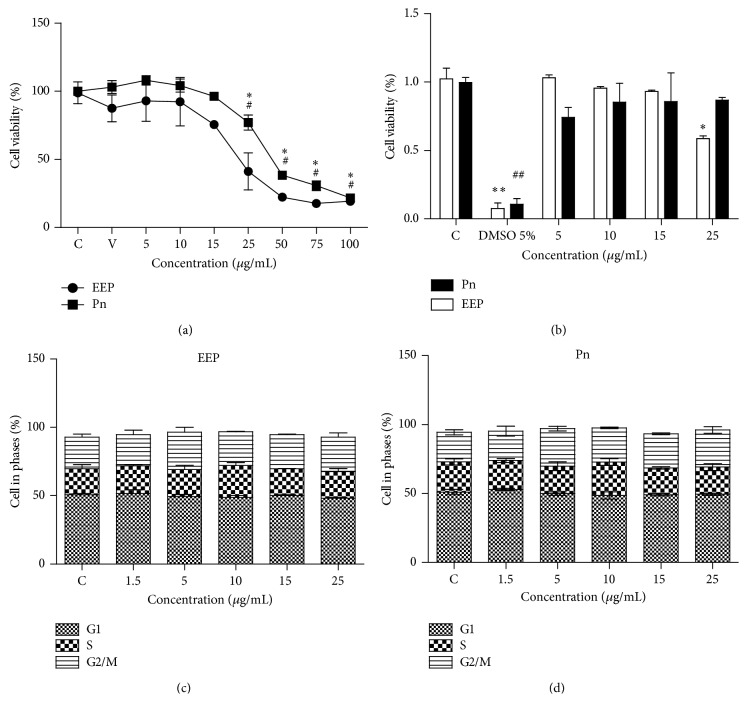
(a) HUVECs viability by MTT assay. (b) Apoptosis/necrosis evaluation by Annexin/PI staining. (c, d) Flow cytometry analysis of cell cycle phases for EEP and Pn treatments, respectively. The data were expressed as mean ± standard deviation. ^*∗*^ *P* < 0.05 in EEP treatment; ^*∗∗*^ *P* < 0.01 in EEP treatment; ^#^ *P* < 0.05 in Pn treatment; ^##^ *P* < 0.01 in Pn treatment. C: control; V: vehicle; Pn: Pinocembrin.

In order to evaluate the proliferating potential and the cell viability of HUVECs exposed to different concentrations of EEP (0–100 *μ*g/mL) or Pn (0–100 *μ*g/mL), the MTT reduction assay and the Annexin V-FITC/PI staining assay were carried out. As shown in Figure 1(a), the treatment with EEP or Pn up to 15 *μ*g/mL did not significantly decrease the cell proliferation assessed with the MTT assay. In addition, treatment with EEP up to 15 *μ*g/mL or Pn up to 25 *μ*g/mL did not induce apoptosis or necrosis Annexin V-FITC/PI (Figure 1(b)). On the other hand, concentration up to 25 *μ*g/mL of EEP or Pn did not induce arrest of the cell cycle (Figures 1(c) and 1(d)). In order to work with no toxic and no apoptotic concentrations, based on this result, we selected concentrations up to 15 *μ*g/mL of EEP or Pn for subsequent experiments.

### 3.2. Endothelial Cells Migration

**Figure 2:**
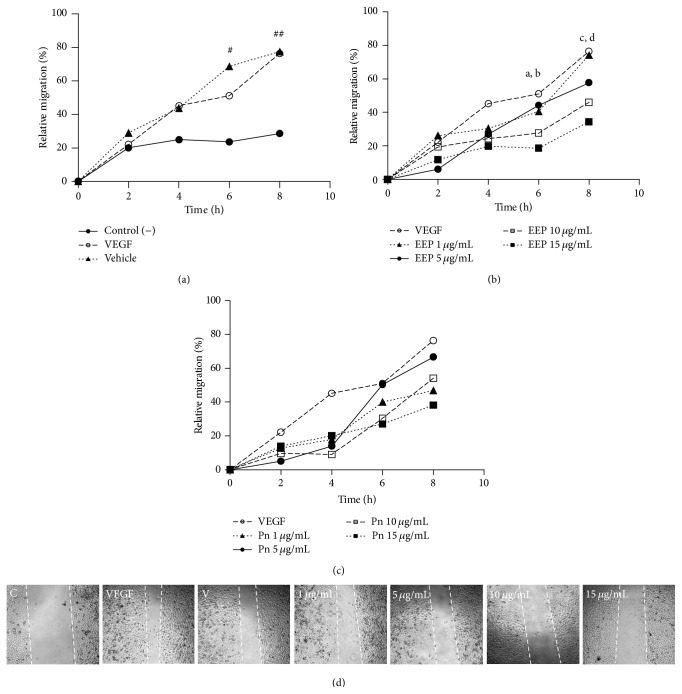
Effect of different concentrations of EEP or Pn on the HUVECs migration at 8 h. (a) Comparison of HUVECs with and without VEGF 10 *µ*g/mL. (b) HUVECs treated with 1–15 *µ*g/mL of EEP. (c) HUVECs treated with 1–15 *µ*g/mL of Pn. (d) Migration in HUVECs treated with 1–15 *µ*g/mL of EEP at 8 h. White lines represent the initial wound. ^#^ *P* < 0.05; ^##^ *P* < 0.01; ^a^ *P* < 0.01 (10 *µ*g/mL versus VEGF); ^b^ *P* < 0.01 (15 *µ*g/mL versus VEGF); ^c^ *P* < 0.01 (10 *µ*g/mL versus VEGF); ^d^ *P* < 0.01 (10 *µ*g/mL versus VEGF). C: control; V: vehicle; Pn: Pinocembrin.

To evaluate possible inhibitory effect of EEP or Pn on HUVECs migration the scratch wound assay was performed. As shown in Figure 2(b), treatment with 10 *μ*g/mL (−39.7%, *P* < 0.01) or 15 *μ*g/mL (−54.9%, *P* < 0.01) of EEP significantly reduced the HUVECs migration at 8 h in a dose-dependent manner (Figure 2(d)) compared to HUVECs treated only with VEGF. By contrast, Pn treatment showed significant changes at different doses (−50.0%, *P* < 0.01 with 15 *μ*g/mL at 8 h); however, the effect was not dose dependent (Figure 2(c)). Importantly, treatment with vehicle did not modify the HUVECs migration (Figure 1(a)).

### 3.3. Tube Formation Assay

**Figure 3:**
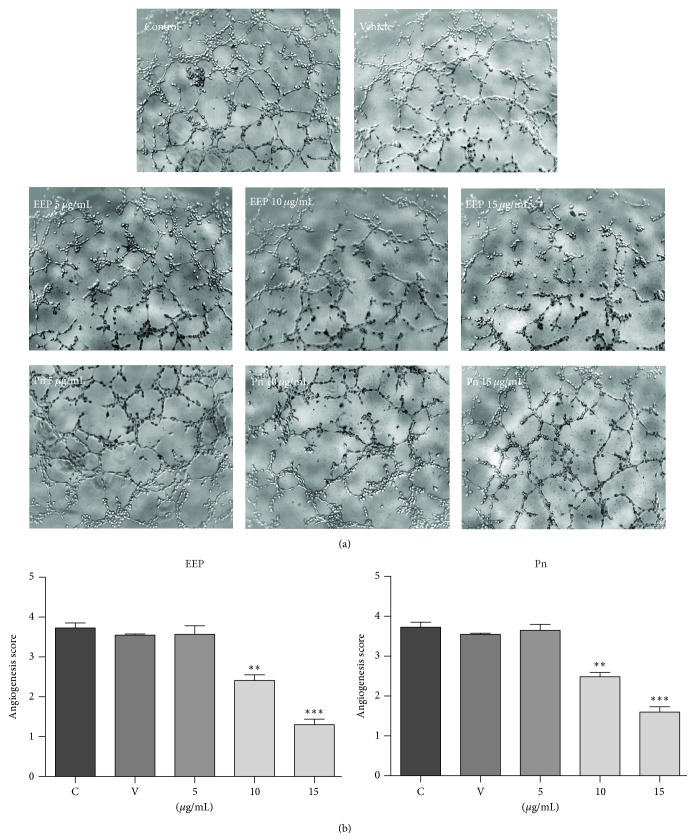
Effect of EPP and Pn on formation of capillary-like structures in Matrigel. (a) Control cells, DMSO (0.1%) treated cells, and EPP or Pn treated cells at 5, 10, and 15 *µ*g/mL, respectively. (b) Angiogenesis score. Data presented as mean ± standard deviation. ^*∗∗*^ *P* < 0.01; ^*∗∗∗*^ *P* < 0.001. C: control; V: vehicle; Pn: Pinocembrin.

The* in vitro *angiogenesis was assessed by capillary-like tube formation assay on Matrigel. Treatment with EEP and Pn had a moderate but significant inhibitory effect on the angiogenesis score (Figure 3(b)) in a dose-dependent manner. Notably, the major effects were in the formation of closed rings of capillary-like structures, an indicator of the ability of HUVECs to form networks (Figure 3(a)).

### 3.4. Aortic Ring Assay

**Figure 4:**
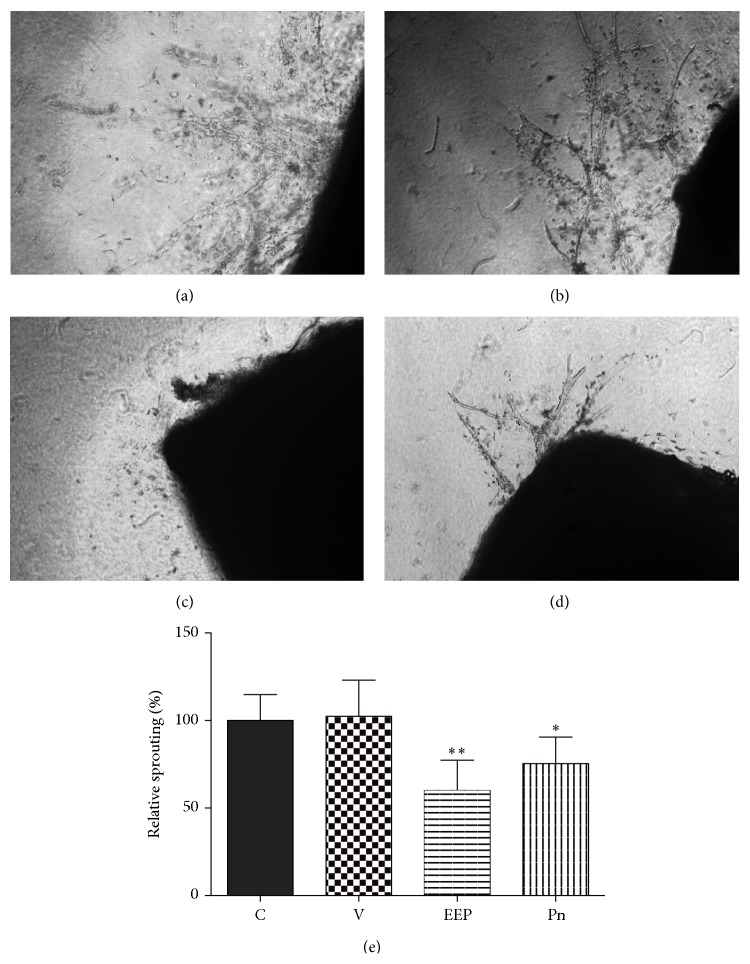
Effect of EEP and Pn on formation of capillary-like structures from aortic ring under VEGF (10 ng/mL) stimulus. (a) Control aortic ring. (b) Aortic ring, DMSO (0.1%) treated. (c) Aortic ring, EEP treated (15 *µ*g/mL). (d) Aortic ring, Pn treated (15 *µ*g/mL). (e) Relative quantification of capillary-like structures. Data presented as mean ± standard deviation. ^*∗*^ *P* < 0.01; ^*∗∗*^ *P* < 0.001. C: control; V: vehicle; Pn: Pinocembrin.

In order to evaluate the effect of EEP or Pn on angiogenesis* ex vivo* the rat aortic ring assay was carried out. At 15 *μ*g/mL both EEP (Figure 4(c)) and Pn (Figure 4(d)) significantly diminished the microvessel sprouting from aortic rings, when compared with control group (Figure 4(a)). Vehicle did not affect microvessel sprouting (Figure 4(b)).

### 3.5. ERK1/2 Phosphorylation and HIF1*α* Stabilization

**Figure 5:**
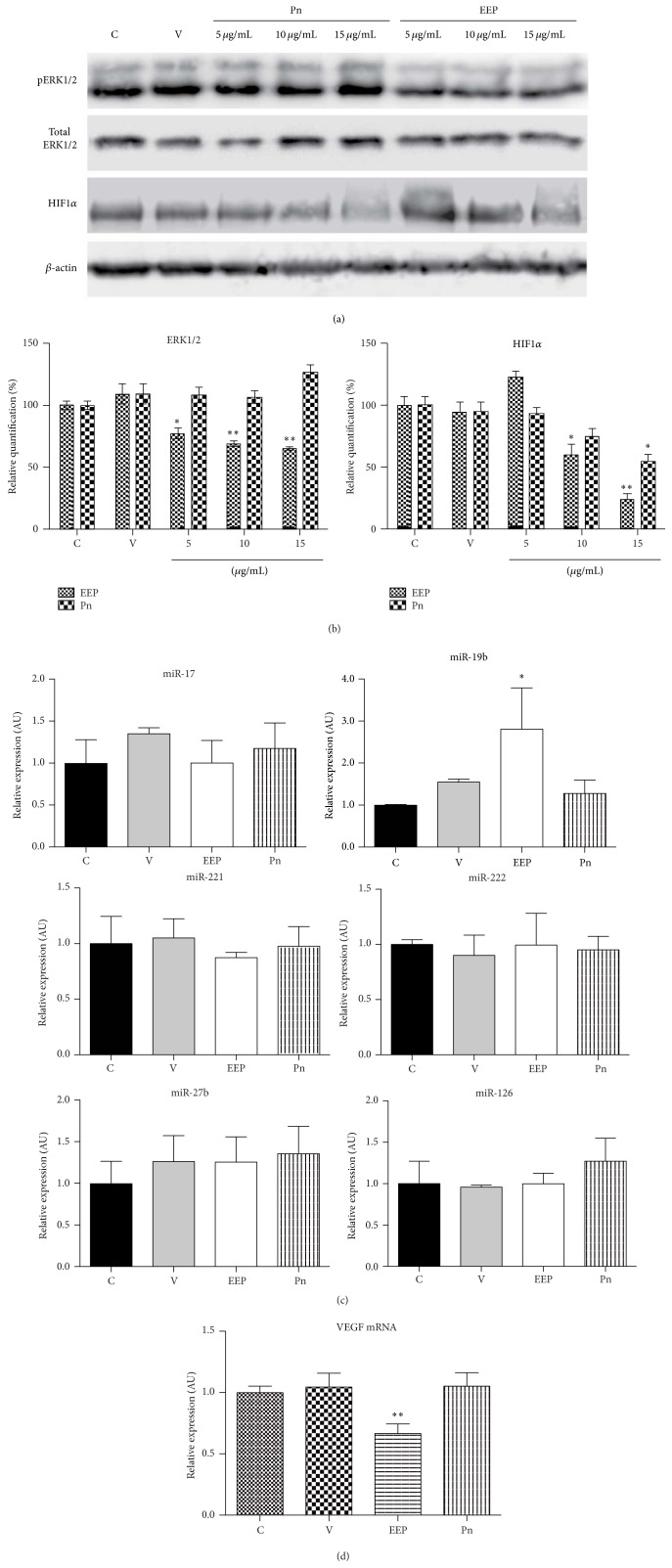
(a) ERK1/2 phosphorylation and HIF1*α* stabilization by western blot. (b) Column bar of quantification of ERK1/2 phosphorylation and HIF1*α* stabilization by western blot. (c) Relative expression of miRNAs associated with angiogenesis. (d) Relative expression of HUVECs mRNA. Bars represent mean ± standard deviation. ^*∗*^ *P* < 0.01; ^*∗∗*^ *P* < 0.001. C: control; V: vehicle; Pn: Pinocembrin.

Western blot analysis was carried out to evaluate the ERK1/2 phosphorylation and the HIF1*α* stabilization, two important factors involved in the induction of angiogenesis. EEP, but not Pn, was able to inhibit slightly the ERK1/2 activation (Figure 5(a)). On the other hand, both EEP and Pn significantly inhibited in a dose-dependent manner the activation of HIF1*α*.

### 3.6. VEGF mRNA and Angiogenesis-Related MicroRNAs Expression

Finally, VEGF mRNA and microRNAs associated with angiogenesis in previous studies (miR-126, miR-19b, miR-221, miR-222, miR-27b, and miR-17) were evaluated by real-time PCR. Only EEP was able to reduce the VEGF mRNA expression (Figure 5(b)). In addition, only miR-19b was overexpressed in HUVECs treated with EEP (Figure 5(c)).

## 4. Discussion

Angiogenesis is a highly regulated process, which involves a complex cascade of events. However, the imbalance of pro- and antiangiogenic factors is able to worsen many pathological conditions like atherosclerosis or cancer. Accumulating evidence has showed that polyphenols can modulate this process [9, 11–13, 25, 27]. In this study, we reported that ethanolic extracts of Chilean propolis and Pinocembrin, one of its main constituents, were able to modulate* in vitro *angiogenesis at no cytotoxic concentration, in part by modulating HIF1*α* stabilization and ERK1/2 phosphorylation, two important factors involved in this process.

We showed that EEP or Pn could modulate* in vitro *HUVECs migration,* in vitro* organization into capillary-like structures, and* ex vivo* formation of new blood vessels. Consistent with our results, previous reports showed a potent inhibitory effect of the propolis extract on capillary-like structures formation of HUVECs, reaching an inhibition between 60% and 90% at 50 *μ*g/mL [28, 29]. It is important to note that many of these studies use higher concentration of propolis extract and fail to clarify whether the* in vitro* effect of propolis is not due to a cytotoxic effect. We showed that concentration above 15 *μ*g/mL of EEP or Pn decreased cell viability, which does not differentiate between a functional effect and a cytotoxic effect.

The inhibitory activity of EEP was more effective than Pn. The suppressing effect of Pn on capillary-like structures formation was weaker than EEP and the migration assay was inconclusive. Phytochemicals, including polyphenols, exert their function mainly by antioxidant or prooxidant activity [30]. Pinocembrin has a lower total antioxidant capacity and reduced free radical-scavenging activity compared with other common polyphenols present in the propolis [31]. Our ethanolic extract of propolis contains over thirty compounds, highlighting Pinocembrin, Pinobanksin-3-O-acetate, and caffeic acid isoprenyl ester [32]. It is possible that because the EEP is a complex mixture the observed effect is due to other compounds with highest antioxidant activity or a synergy between multiple compounds.

In accordance with our results, previous studies have showed that polyphenols of propolis can modulate HIF1*α* and ERK1/2 in endothelial cells [25, 33]. HIF1*α* is a transcription factor that responds to low concentrations of oxygen in the cellular environment. Under hypoxic conditions, HIF1*α* is stabilized and translocated to nucleus to induce angiogenic factors, such as VEGFA, a major contributor to angiogenesis. VEGFA/VEGFR2 signaling induces angiogenesis by cell proliferation, survival, and migration in part through the activation of the mitogen-activated protein kinase/extracellular-signal-regulated kinase-1/2 (ERK1/2) and phosphatidylinositol 3-kinase (PI3-K)/Akt signal transduction pathways [34]. In line with this, we showed that EEP could inhibit the HIF1*α* accumulation and the ERK1/2 phosphorylation in a dose-dependent manner, which is related with the suppression of VEGF mRNA and is consistent with the antiangiogenic effect demonstrated in the functional assays.

Finally, we conducted a small microRNA screening that has been associated with angiogenesis in previous studies. Among them, only miR-19b was overexpressed in cells treated with EEP.* In silico* and* in vitro* analyses have suggested that miR-19b targets mRNA corresponding to the proangiogenic proteins FGFR2 and MAPK1 (ERK2). In addition, previous work showed that miR-19b blocks the cell cycle from the S phase to the G(2)/M phase transition by controlling the expression of cyclin D1c [35].

## 5. Conclusion

In summary, the findings in the current study demonstrate that a nonapoptotic/toxic concentration of polyphenol-rich extract of Chilean propolis can modulate* in vitro* angiogenesis in part by modulating HIF1*α* and ERK1/2 signaling pathway and mechanism involving miR-19b. The effect showed by EEP was not completely replicated by Pn, demonstrating the importance of the combined action of multiple compounds from an extract; however, more studies should be accomplished.
